# Deletion of Sigmar1 leads to increased arterial stiffness and altered mitochondrial respiration resulting in vascular dysfunction

**DOI:** 10.3389/fphys.2024.1386296

**Published:** 2024-04-29

**Authors:** Naznin Sultana Remex, Chowdhury S. Abdullah, Richa Aishwarya, Gopi K. Kolluru, James Traylor, Mohammad Alfrad Nobel Bhuiyan, Christopher G. Kevil, A. Wayne Orr, Oren Rom, Christopher B. Pattillo, Md. Shenuarin Bhuiyan

**Affiliations:** ^1^ Department of Molecular and Cellular Physiology, Louisiana State University Health Sciences Center-Shreveport, Shreveport, LA, United States; ^2^ Department of Pathology and Translational Pathobiology, Louisiana State University Health, Shreveport, LA, United States; ^3^ Department of Internal Medicine, Louisiana State University Health Sciences Center-Shreveport, Shreveport, LA, United States

**Keywords:** SIGMAR1, physiological function, vascular function, mitochondria, flow mediated dilatation (FMD)

## Abstract

Sigmar1 is a ubiquitously expressed, multifunctional protein known for its cardioprotective roles in cardiovascular diseases. While accumulating evidence indicate a critical role of Sigmar1 in cardiac biology, its physiological function in the vasculature remains unknown. In this study, we characterized the expression of Sigmar1 in the vascular wall and assessed its physiological function in the vascular system using global Sigmar1 knockout (Sigmar1^−/−^) mice. We determined the expression of Sigmar1 in the vascular tissue using immunostaining and biochemical experiments in both human and mouse blood vessels. Deletion of Sigmar1 globally in mice (Sigmar1^−/−^) led to blood vessel wall reorganizations characterized by nuclei disarray of vascular smooth muscle cells, altered organizations of elastic lamina, and higher collagen fibers deposition in and around the arteries compared to wildtype littermate controls (Wt). Vascular function was assessed in mice using non-invasive time-transit method of aortic stiffness measurement and flow-mediated dilation (FMD) of the left femoral artery. Sigmar1^−/−^ mice showed a notable increase in arterial stiffness in the abdominal aorta and failed to increase the vessel diameter in response to reactive-hyperemia compared to Wt. This was consistent with reduced plasma and tissue nitric-oxide bioavailability (NOx) and decreased phosphorylation of endothelial nitric oxide synthase (eNOS) in the aorta of Sigmar1^−/−^ mice. Ultrastructural analysis by transmission electron microscopy (TEM) of aorta sections showed accumulation of elongated shaped mitochondria in both vascular smooth muscle and endothelial cells of Sigmar1^−/−^ mice. In accordance, decreased mitochondrial respirometry parameters were found in *ex-vivo* aortic rings from Sigmar1 deficient mice compared to Wt controls. These data indicate a potential role of Sigmar1 in maintaining vascular homeostasis.

## 1 Introduction

Sigma1 receptor protein (Sigmar1) is a small molecular chaperone protein that is composed of 223 amino acids with a molecular mass of 25.3 KDa (Kekuda et al., 1996; Seth et al., 1997; Seth et al., 1998; Mei and Pasternak, 2001). This protein has a single putative transmembrane domain, and the Sigmar1 gene consists of four exons and three introns with exon 3 being the shortest and exon 4 being the longest (Prasad et al., 1998). Sigmar1 was first identified as a subtype of opioid receptors using specific radiolabeled ligand binding assays (Martin et al., 1976), and later, it was cloned from guinea pig, rat, and mouse tissues (Hanner et al., 1996; Kekuda et al., 1996; Seth et al., 1997; Seth et al., 1998). Human and animal studies have identified ubiquitous expression of Sigmar1 in almost all the body tissues, including brain, heart, liver, lung, kidney, skeletal muscle, stomach, spleen, thymus, and other regions (Kekuda et al., 1996; Mei and Pasternak, 2001). Even though Sigmar1 is ubiquitously expressed in almost all body tissues, exploring its pathophysiological role in different organ systems has so far been limited to the brain, heart, kidney, and few other organs.

Sigmar1 has been extensively studied in the central nervous system, and the association of SIGMAR1 gene mutation has been demonstrated in numerous neurodegenerative diseases, such as, Parkinson’s disease, Alzheimer’s disease, Huntington’s disease, silver-like syndrome, amyotrophic lateral sclerosis, and certain psychiatric disorders (Mishina et al., 2005; Mishina et al., 2008; Aishwarya et al., 2021). Several studies from our group and others have uncovered Sigmar1’s involvement in cardiac function. Sigmar1 global knockout (Sigmar1^−/−^) mice showed cardiac hypertrophy, contractile dysfunction, perivascular and interstitial fibrosis in the heart, and a decline in mitochondrial respiratory function (Abdullah et al., 2018). Activating Sigmar1 using its non-selective ligand showed a protective role in heart failure and pressure-overload-induced cardiac injury models of rats and mice (Bhuiyan and Fukunaga, 2009; Bhuiyan et al., 2010; Tagashira et al., 2010; Bhuiyan and Fukunaga, 2011). The ultrastructural analysis of the hearts from Sigmar1^−/−^ mice showed accumulation of elongated-shaped mitochondria consistent with alteration in mitochondrial respiratory parameters and bioenergetics, which likely contributes to the impaired cardiac contractile function in the absence of Sigmar1 (Abdullah et al., 2018).

The subcellular distribution of Sigmar1 is highly tissue and cell-type dependent. It was initially thought that Sigmar1 is specifically localized in the mitochondria-associated endoplasmic reticulum membrane (MAM). However, later studies revealed that Sigmar1 is also present in the plasma membrane, ER, nuclear envelope, and in the lysosome (Dussossoy et al., 1999; Hayashi and Su, 2007; Su et al., 2009). The presence of Sigmar1 in the mitochondria was first identified by Klouz et al., 2002 in 2002 in the liver tissue section using colocalization of Sigmar1 with a mitochondrial marker. Recent studies confirmed Sigmar1’s localization in the mitochondria of cardiomyocytes using quantum dot-mediated immunolabeling of Sigmar1 using transmission electron microscopy (TEM), co-immunostaining with mitochondria-specific MitoTracker, and using subcellular fractionation assays (Abdullah et al., 2022a; Aishwarya et al., 2022a). Thus, Sigmar1 has a potential role in regulating mitochondrial respiration and bioenergetics that is essential to continue important cellular functions.

The existence of Sigmar1 protein in the vasculature of the thoracic aorta in rats was reported using Western blot analysis (Bhuiyan and Fukunaga, 2009). In pressure overload-induced cardiac injury and transverse aortic constriction (TAC) models, the expression of Sigmar1 was found to be significantly decreased in the thoracic aorta, leading to vascular remodeling. This reduced Sigmar1 expression was further associated with downregulation of Akt-eNOS signaling axis in the aorta, which was rescued by activating Sigmar1 using its ligands, e.g., DHEA and fluvoxamine (Bhuiyan and Fukunaga, 2009; Bhuiyan et al., 2010; Tagashira et al., 2010; Bhuiyan et al., 2011a; Bhuiyan et al., 2011b; Tagashira et al., 2011; Tagashira et al., 2013). This suggested the potential role of Sigmar1 in vascular system and remodeling. However, only a few studies to date have examined the role of Sigmar1 in the vasculature.

Activation of Sigmar1 using its agonist improved the barrier function of vascular endothelial cells and decreased its permeability to albumin and dextran (Motawe et al., 2020). Similarly, the Sigmar1 pharmacologic ligand, PRE084, enhanced endothelial integrity and barrier function after inflammatory induction in lymphatic endothelial cells (Motawe et al., 2021a). These studies also demonstrated Sigmar1’s involvement in mitochondrial bioenergetics regulation in vascular and lymphatic endothelial cells. A recent study showed that the opioid receptor agonist, oxycodone, preserves cardiac microvascular endothelial cell integrity and myocardial function after ischemia-reperfusion injury by binding to Sigmar1 (Ji et al., 2022). Pharmacologic inhibition of Sigmar1 using its antagonist, such as haloperidol metabolite-II valproate ester [(±)-MRJF22] exhibited an antiangiogenic effect, significantly reducing cell viability, migration, and tube formation in human retinal endothelial cells stimulated with vascular endothelial growth factor A (Olivieri et al., 2016). However, these previous studies were based only on the use of pharmacological ligands, and lacked genetic manipulation of Sigmar1 to establish its physiological roles in the vascular system.

The current study aims to explore the molecular and physiological roles of Sigmar1 associated with vascular tone, vascular remodeling, mitochondrial structure, and function using genetic mouse models. We used gender-matched Sigmar1 global knockout (Sigmar1^−/−^) and wild-type littermate control (Wt) mice for all our experiments.

## 2 Materials and methods

### 2.1 Animals and human tissues

Both male and female age-matched wildtype (Wt) control and Sigmar1 global knockout mice (Sigmar1^−/−^) on C57BL6 background were used for this study (Bernard-Marissal et al., 2015; Abdullah et al., 2018). Mice between the ages of 3–4 months were used for all the experiments for this study to understand the physiological role of Sigmar1 protein in the vascular system using a loss of function approach. All animals were housed in a temperature-maintained, well-controlled environment with a 12 h light-dark cycle and provided regular chow-diet *ad libitum* food and water. All experimental procedures and protocols for using laboratory animals were approved by the Institutional Animal Care and Use Committee at Louisiana State University Health Sciences Center-Shreveport. The animals were cared for and handled according to the Guide for the Care and Use of Laboratory Animals (Care IoLARCo and Animals UoL, 1986). The human left anterior descending coronary artery (LAD) tissues were obtained from individuals’ hearts during autopsy and fixed in formalin obtained in collaboration with the Department of Pathology and Translational Pathobiology at Louisiana State University Health Sciences Center-Shreveport. The LAD tissue used in this manuscript was collected from a 47 years old white male with no apparent history of cardiovascular complications and the cause of death was bronchial pneumonia. Human LAD tissues used in this study were deemed non-human research by the local IRB owing to the exclusive use of *postmortem* samples.

### 2.2 Histology staining

3–4 months-old Wt and Sigmar1^−/−^ mice were euthanized using isoflurane overdose, and hearts were immediately harvested. Hearts with aortic roots were dropped in 4% buffered formalin for fixation for at least 24 h, processed, and embedded in the paraffin. Then the paraffin blocks were cut into 5 μm thin serial sections, and the same sections were used for histological staining to compare between groups. The vascular reorganization was assessed by hematoxylin and Eosin (H&E) staining using H&E kit (H-3502, Vector Laboratories) following the manufacturer’s protocol. Vascular and perivascular collagen deposition was analyzed by Masson’s trichrome, Russell-movat pentachrome, and toluidine blue staining of aortic roots following a previously published protocol (Yurdagul et al., 2014; Molnar et al., 2017; Remex et al., 2023). All stained tissue sections were imaged under ×10 objective lens using an Olympus BX40 brightfield microscope in an investigator-blinded manner. Tissue sections from both male and female mice from each genotype were analyzed (N = 3 mice per group, Wt; 1 male and 2 female mice, and Sigmar1^−/−^; 2 males and 1 female mice).

### 2.3 Immunohistochemistry

Formalin-fixed and paraffin-embedded tissue sections of human and mouse blood vessels were used to see Sigmar1 protein expression using immunohistochemical staining following previously used methods (Abdullah et al., 2020). Briefly, 5 μm thin tissue sections were deparaffinized, rehydrated, and used for antigen retrieval using antigen unmasking solution (H-3301, Vector Laboratories, Burlingame, CA). The sections were then blocked for endogenous peroxidases using 0.3% v/v hydrogen peroxide and with a blocking reagent containing 5% serum after that at room temperature (Vector Laboratories, Burlingame, CA). Next step was primary antibody incubation overnight at 4°C (Sigmar1 #61994S, phos-eNOS^ser1177^ #9571S, Cell Signaling Technology, Danvers, MA, dilution 1:100). Next day, the tissue sections were incubated with secondary antibody at room temperature for 1 h 30min in a humidified slide box. Vectastain Elite Peroxidase Kit (Vector Laboratories, Burlingame, CA) was used to amplify the signal between antigen and antibodies for visualization. A highly sensitive Dako DAB Chromogen system (DAKO, Carpinteria, CA) was used following manufacturer’s protocol to precipitate brown color at the sites of the target antigen. Aortic root tissue sections were then counterstained with hematoxylin, washed in ethanol, dehydrated, and mounted using Cytoseal XYL mounting medium (Thermo Fisher Scientific, Waltham, MA). They were left for at least 4–8 h to dry, and images were taken using an Olympus BX40 brightfield microscope under ×10 objective lens using Olympus cellSens imaging software (Olympus Life Science, Waltham, MA). Images were taken in an investigator-blinded fashion, and the DAB-positive brown precipitated areas on the tissue sections were considered antibodies-positive areas.

### 2.4 Immunofluorescence

5 μm thin sections of mouse aortic roots and human LAD tissues were deparaffinized and rehydrated. This was followed by antigen retrieval using antigen unmasking solution of 10 mmol/L sodium citrate buffer of pH 6.0 by boiling at 100°C for 30 min in a conventional microwave at power level 1. The tissue sections were then blocked for an hour at room temperature using blocking buffer (1% bovine serum albumin, 0.1% fish skin gelatin in cold water, and 1% tween 20 in PBS). Primary antibodies incubation overnight at 4°C (Sigmar1 #61994S, Cell Signaling Technology, Danvers, MA, dilution 1:100) was followed by Alexa fluor-conjugated secondary antibodies incubation at room temperature for 1 h 30 min (dilution 1:100) in the humidified slide stain box. Tissue sections were then counterstained with nuclear DAPI (Invitrogen, Waltham, MA) for 5 min, washed, and mounted using Vectashield Hardest Antifade mounting media (H1400; Vector Laboratories, Burlingame, CA). Tissue slides were placed in a dark place for at least 4–8 h before imaging. Images were taken under ×10 objective lens using a Nikon A1R high-resolution confocal microscope with Nikon NIS elements software (v.4.13.04).

### 2.5 Pulse wave doppler velocity

Pulse wave Doppler velocity (PWV) was measured as an index of vascular stiffness in the aorta of Wt and Sigmar1^−/−^ mice at 3 months of age using male mice (N = 7 Wt and 8 Sigmar1^−/−^ mice). This is a non-invasive indirect determination of arterial rigidity *in-vivo* (Williams et al., 2007; DuPont et al., 2021). Briefly, Wt and Sigmar1^−/−^ littermate control mice were anesthetized and positioned on a warm ultrasound table and observed to have stable heart and respiration rates. A Vevo 3100 ultrasound probe was aligned over the mice aorta to record pulse wave Doppler ultrasound velocity measurements, coinciding with the pressure upstroke in mice. This was performed at a proximal and distal location, and the echocardiogram (ECG) signals were recorded. The transit time was calculated from the difference between distal and proximal arrival times at these two locations on the aorta. Finally, the PWV was determined by dividing the distance between two locations (measured from B-mode images) by the transit time. The data was analyzed using VEVO LAB analysis software.

### 2.6 Flow-mediated dilation

We followed a previously published non-invasive method of flow-mediated dilation (FMD) of the left femoral artery to measure vascular function in mice *in-vivo* (Schuler et al., 2014; Pardue et al., 2020; Kolluru et al., 2022). Both male and female Wt and Sigmar1^−/−^ mice were subjected to FMD at 4 months of age (N = 5 Wt and 6 Sigmar1^−/−^ mice). Briefly, mice were anesthetized using isoflurane, and hairs from the hindlimb were removed using hair-removing lotion. The mice were then placed on an ultrasound table that maintained a constant 37°C temperature and was equipped with ECG. A vascular occluder cuff of 5 mm diameter (Harvard Apparatus) was placed around the left proximal hindlimb of mice, and the Doppler ultrasound probe (VEVO 3100, VisualSonics) was manually aligned over the femoral artery distal to the occluding cuff. Vessel diameter was measured using M mode at the baseline level before introducing the ischemic trigger. Transient vascular occlusion was induced in the distal hindlimb by inflating the occlusion cuff manually with an air-filled syringe for 1 min. Then, the cuff was deflated to remove the trigger, and the left femoral arterial diameter was measured for 300 s at 30-s intervals. The recorded data were analyzed using VEVO LAB analysis software.

### 2.7 Nitric oxide (NOx) measurement

NO and its metabolites (NOx) were calculated in the plasma and in one of the skeletal muscles (gastrocnemius) of Wt and Sigmar1^−/−^ mice. Both male and female Wt and Sigmar1^−/−^ mice at 4 months of age were used for this experiment (N = 4; 2 males and 2 females per group). A previously described ozone-based very sensitive chemiluminescent assay (Sievers Nitric Oxide Analyzer 280i, Weddington, NC) was used to measure total NOx (Kolluru et al., 2015; Kolluru et al., 2022). Briefly, blood was collected from mice using cardiac puncture, and plasma was isolated by centrifugation. Isolated plasma and the gastrocnemius muscle samples were immediately collected in NO preservation buffer that contained 1.25 mol/L potassium ferricyanide, 56.9 mmol/L N-ethylmaleimide, and 6% Nonidet P-40 substituted in phosphate buffer saline (PBS) solution. Aliquots of plasma samples went through a sulfanilamide or mercuric chloride resistance test following a reaction with an acidic sulfanilamide solution or mercuric chloride by 0.5% v/v and waited in the dark for 15 min. Samples were then injected into the analyzer to measure the total NOx.

### 2.8 Protein isolation and Western blot

Total proteins from the tissue were extracted from Wt and Sigmar1^−/−^ mice aorta and prepared for Western blot to examine protein expression levels in the tissue following the previous protocol (Abdullah et al., 2018; Remex et al., 2023). The whole aorta (aortic arch, thoracic, and abdominal aorta) was harvested from the mice and immediately snap-frozen. The tissues were lysed using an ice-cold Cell lytic M buffer (Sigma-Aldrich, St. Louis, MO) supplemented with a complete cocktail of protease and phosphatase inhibitors (Roche, Basel, Switzerland). Using a bead homogenizer with glass beads (Precellys Lysing Kit #03961-1-204, Bertin Technologies, Paris Region, France), we homogenized the aorta tissues and sonicated the homogenized aorta lysates. The whole lysed material was then centrifuged to separate the soluble proteins in the supernatant from the insoluble cell debris in the precipitate. We took the supernatant out, measured protein concentrations using a modified Bradford Reagent (Bio-Rad Laboratories, Hercules, CA), and prepared an equal amount of proteins in 6x Laemmli’s sample buffer for Western blotting. For protein separation according to the molecular weights, about 20–30 μg of proteins were used to run through the sodium dodecyl sulfate-polyacrylamide gel electrophoresis (SDS-PAGE) and transferred to polyvinylidene difluoride (PVDF) membrane (Bio-Rad Laboratories, Hercules, CA). The transfer efficiency was checked using a protein stain dye, Ponceau S (Acros Organics, Geel, Belgium), which could also be used as a loading control. The transferred proteins on the PVDF membrane were then blocked using 5% non-fat skim milk at room temperature on the rocker for 1 h and incubated with primary antibodies overnight on slow rocking in the cold room at 4°C. The secondary antibodies’ incubation was at room temperature for 1 h and 30 min on the rocker. The secondary antibodies were conjugated with alkaline phosphatase (Jackson ImmunoResearch Laboratories, Inc., West Grove, PA) that was developed and detected by ECF substrate (Amersham, Little Chalfont, United Kingdom) and imaged using a ChemiDoc Touch Imaging System (Bio-Rad Laboratories, Hercules, CA). Bio-Rad Image Lab software v.6.0.1 was used to visualize the scanned images, and densitometric analysis was performed in NIH ImageJ software v1.6.0 (Bethesda, MD). The primary antibodies that were used in this study were- Sigmar1 (#61994S, Cell Signaling Technology, Danvers, MA; dilution 1:1000), phos-eNOS^ser1177^ (#9571S, Cell Signaling Technology, Danvers, MA, dilution 1:1000), eNOS (#32027S, Cell Signaling Technology, Danvers, MA; dilution 1:1000), and β-Actin (#sc-47778, Santa Cruz Biotechnology, Santa Cruz, CA; dilution 1:10000).

### 2.9 Transmission electron microscopy

Transmission electron microscopy (TEM) was performed to see ultrastructure of mouse aorta tissue following the previously described method (Su, 1982; Alam et al., 2018). Briefly, we isolated aorta from Wt and Sigmar1^−/−^ mice at 4 months of age (N = 4 mice; 2 males and 2 females per group), immediately cut them into small 1 mm pieces, and left them in a buffer for overnight fixation that contains 3% glutaraldehyde in 0.1 mol/L sodium cacodylate buffer (pH 7.2). The tissues then underwent a post-fixation step using 1% osmium tetroxide (OsO_4_) solution, followed by processing for tissue sectioning. The small pieces of aorta tissue were counterstained with uranyl acetate and lead salts before embedding in epoxy resins with low viscosity. The embedded tissue blocks were then cut into ultra-thin sections and stained for toluidine blue to check the quality of ultrastructural organizations of the aortic sections, confirming tissue integrity. The final step was to image the sections using an advanced microscope-compatible digital camera (Woburn, MA) under JEOL-JEM-1400 TEM (JEOL, Peabody, MA). Images from Wt and Sigmar1^−/−^ mice aorta tissues were analyzed to visualize the ultrastructure of both endothelial and vascular smooth muscle cells. The analysis was done in an investigator blinded manner.

### 2.10 High-resolution respirometry

To assess mitochondrial respiratory functions in mouse aorta, we conducted high-resolution respirometry using Oroboros Oxygraph-2k (O2k; Oroboros Instruments, Innsbruck, Austria) (Tyrrell et al., 2020; Abdullah et al., 2022a). The method was adapted and modified from a previously published research article to measure mitochondrial respiration in aortic rings isolated from Wt and Sigmar1^−/−^ mice (Tyrrell et al., 2020). Aortas from the aortic root to the bifurcation of iliac arteries (aortic arch, and thoracic and abdominal aorta) were isolated from 3 months-old mice (N = 5 mice; 3 males and 2 females per group), perivascular fats were carefully removed, and placed in freshly prepared ice-cold respiration medium comprising mannitol/sucrose-EGTA buffer containing 225 mmol/L mannitol, 75 mmol/L sucrose, 5 mmol/L HEPES, and 1 mmol/L EGTA (pH 7.4). The aortas were then cut into small rings (2–3 mm), placed in 2 mL of MiR05 respiration media (60101–01, Oroboros Instruments), and subjected to respirometry studies in O2k respiration chambers at 37°C. Oxygen fluxes were measured following the sequential addition of mitochondrial fuel substrates, uncoupler, and inhibitors of electron transport chain complexes. Instrumental and chemical oxygen background fluxes were calibrated as a function of oxygen concentration and subtracted from the total volume-specific oxygen flux according to the manufacturer’s instructions (Datlab Software, Oroboros Instruments). The following substrates and inhibitors for mitochondrial oxidative phosphorylation (OXPHOS) complexes were sequentially injected into the respiration chambers: 4 mM pyruvate, 4 mM malate, 10 mM glutamate, 2 mM ADP, 5 μM cytochrome C to check mitochondrial membrane integrity, 20 mM succinate, 1.5 μM FCCP, 1 μM Rotenone, and 2.7 μM Antimycin A. The polarographic oxygen flux measurements were recorded every 3-s interval until a steady-state respiration rate was obtained. The respiration rate was calculated from a minimum of 30 data points at the steady state rate and normalized with the wet weight of the aorta tissue. The unit for oxygen flux was expressed as picomoles O_2_ per second per mg of tissue (pmoles/sec/mg). N = 5 mice per group, including both males and females and the data were analyzed in an investigator-blinded manner.

### 2.11 Statistical analysis and data reproducibility

All statistical analyses were performed and graphs were generated using GraphPad Prism version 8 (La Jolla, CA). Normality distribution of the data sets were checked using Kolmogorov-Smirnov test. The data sets that passed normality assumption test were analyzed using unpaired Student’s t-test for groups of two. The flow-mediated dilation data were analyzed using two-way ANOVA with repeated measures on the second factor followed by Sidak’s multiple comparisons. The data sets that failed the normality assumption test were analyzed using non-parametric Mann-Whitney U test and Kruskal-Wallis tests. A *p*-value of equal or less than *0.05* was considered as statistically significant for all tests. All data were represented as bar graphs with individual value points and error bars were represented as mean ± standard error of the mean (SEM). All *in-vivo* experiments were performed in an investigator blinded manner and animal selection for each group or genotype was completely random. A numerical ear tagging system was used for the animals used in the experiments to avoid biasness of the data collection. All the imaging analyses used alphanumerically labeled paraffin blocks, tissue slides, and microscopic images. The image identifier numbers for each animal were cross-checked and matched before proceeding with the analyses.

## 3 Results

### 3.1 Sigmar1 expression in the vascular wall of human and mouse blood vessels

**FIGURE 1:**
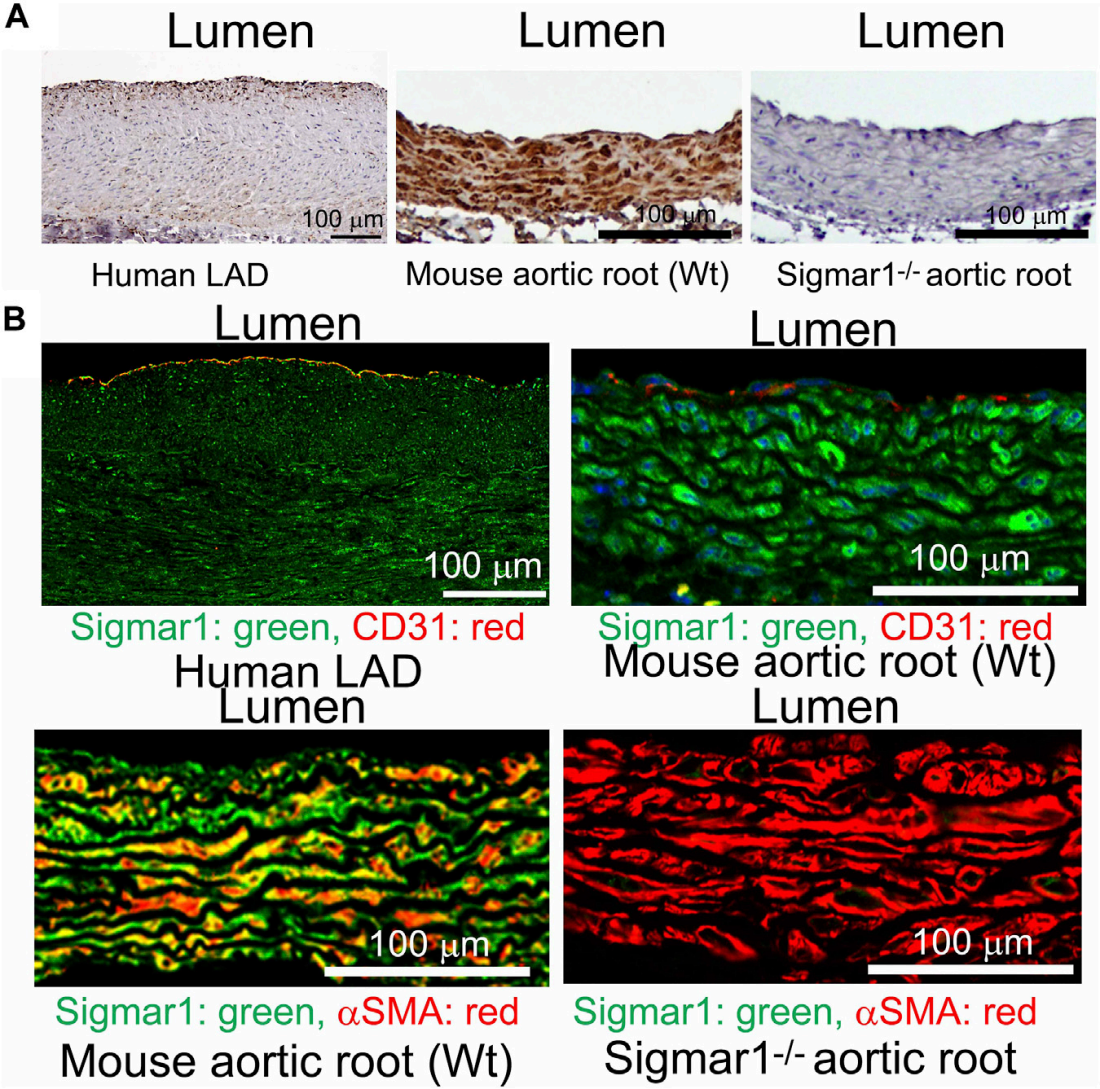
Sigmar1 is expressed in the vascular walls of mouse and human blood vessels. Paraffin-fixed mouse aortic roots and human left anterior descending coronary artery (LAD) tissue sections were used for immunohistochemical and immunofluorescence staining to determine the protein expression level of Sigmar1 in the vasculature. **(A)** immunohistochemical staining of human LAD and mouse aortic root tissue sections from wildtype mice (Wt) using anti-Sigmar1 antibody showing Sigmar1 protein expression in all three vascular layers-intima, media, and adventitia (Shown in brown precipitates). The aortic root tissue section from the Sigmar1 global knockout mouse (Sigmar1^−/−^) was used as a negative control for showing the specificity of anti-Sigmar1 antibody. **(B)** Immunofluorescent staining of human LAD and mouse aortic roots showing Sigmar1 protein expression in the blood vessel layers. Anti-Sigmar1 (green) co-immunostained with endothelial marker CD31 (red) or smooth muscle cell marker αSMA (red). Negative staining for Sigmar1 in the Sigmar1^−/−^ aortic root was used as a negative control. Images were taken from two biological replicates, and at least 10 to 12 microscopic fields were analyzed. N = 2 mice per group (1 male and 1 female per group). Scale bar 100 μm.

We previously reported the expression of Sigmar1 protein in the thoracic aorta lysates of rats in a pressure-overload-induced cardiac injury model using Western blot (Bhuiyan and Fukunaga, 2009). However, Sigmar1’s localization in different cells in the vascular wall lining has yet to be explored. In this study, we used human and mouse blood vessels to show Sigmar1’s expression in the vascular cells. We used formalin-fixed and paraffin-embedded human left anterior descending coronary artery (LAD) and mouse aortic root tissue sections to characterize Sigmar1 protein expression using immunohistochemical (IHC) staining. Brown precipitation from IHC (Figure 1A) showed that Sigmar1 is substantially expressed in both human and mouse arteries in vascular intima, media, and adventitial layers. A negative staining from global Sigmar1 knockout (Sigmar1^−/−^) mouse aorta confirmed the specificity of the Sigmar1 antibody used for IHC staining. Paraffin-fixed tissue sections of human LAD and mouse aortic roots were also used for immunofluorescence (IF) staining to delineate Sigmar1’s expression in all three layers of the vascular wall (green), including the innermost endothelial lining and medial vascular smooth muscle cells (Figure 1B). Both IHC and IF staining results confirmed the expression of Sigmar1 protein in the vascular wall at substantial density.

### 3.2 Role of Sigmar1 in extracellular matrix remodeling and fibrosis in the vascular wall

**FIGURE 2:**
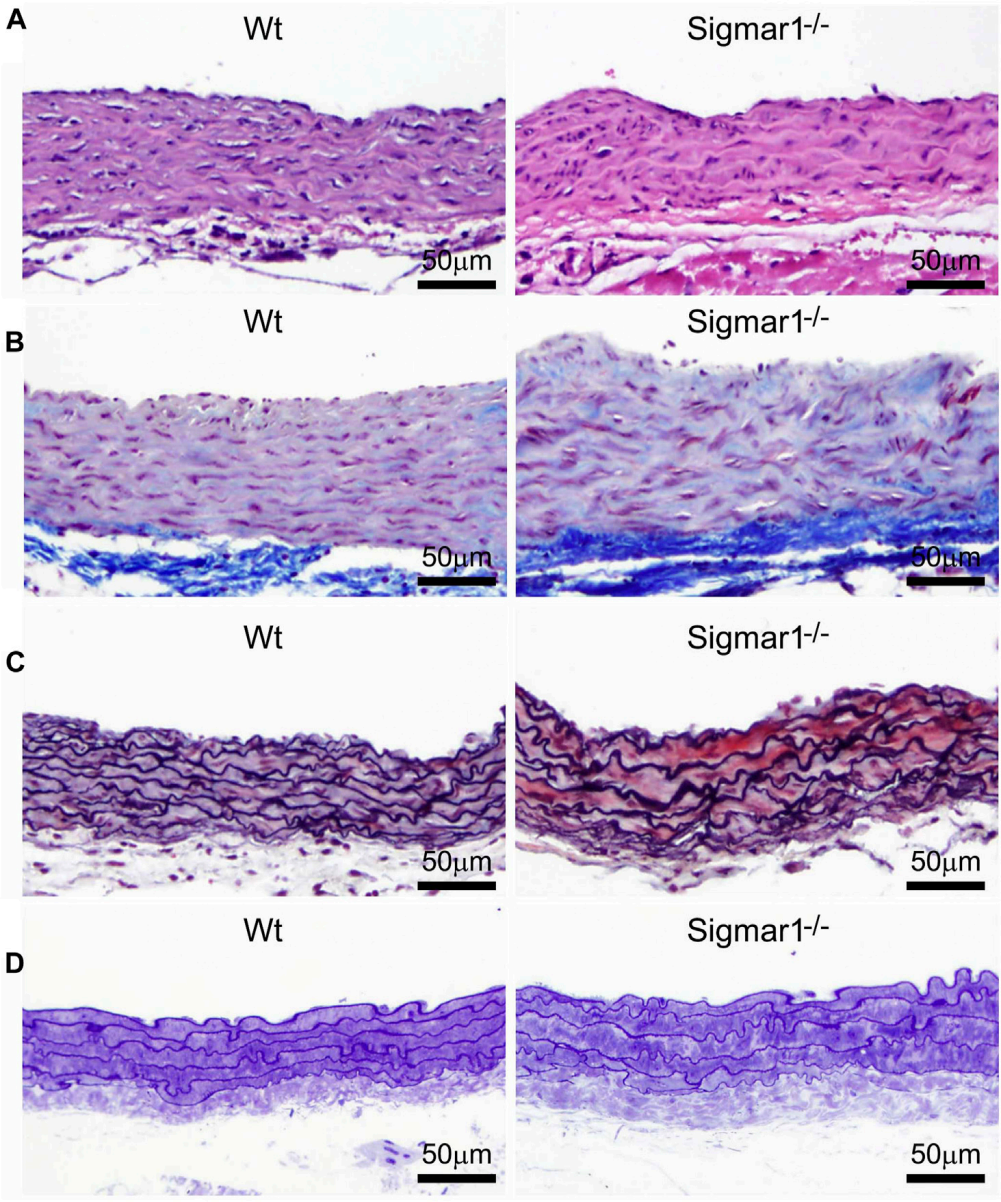
Sigmar1 modulates extracellular matrix remodeling and vascular reorganization in the blood vessel wall. Histological staining of paraffin-fixed mouse aortic root tissue sections visually represents blood vessel walls from wildtype (Wt) and Sigmar1^−/−^ mice. **(A)** Hematoxylin and Eosin (H&E) staining of aortic root showing reorganization of smooth muscle cell layer determined by nuclei disarray and thick extracellular matrix (ECM) in the adventitial layer in the absence of Sigmar1 compared to Wt. **(B)** Masson’s trichrome staining of mouse aortic roots in the Sigmar1^−/−^ mice shows a remarkably higher amount of collagen deposition in the ECM and medial layer disarray. **(C)** Russell-Movat pentachrome staining of formalin-fixed aortic root tissue sections shows abrupt organization of elastic lamina in the medial layer and higher fibrosis in the Sigmar1^−/−^ mice blood vessel wall. **(D)** Toluidine blue staining of aortic root microsections showing altered blood vessel reorganizations and thick ECM layer in the Sigmar1^−/−^ mice aortic root, indicating Sigmar1’s potential role in vascular tissue reorganization and ECM remodeling. Aortic root tissues of both genotypes were collected from male and female littermate mice between the ages of 3–4 months-old. N = 3 mice per group (Wt; 1 male and 2 female mice, Sigmar1^−/−^; 2 males and 1 female mice). At least 10 to 12 microscopic fields per sample were analyzed in an investigator-blinded fashion. Scale bar 50 μm.

Previous studies provided evidence of extracellular matrix remodeling and collagen deposition leading to fibrosis in different organs of Sigmar1^−/−^ mice, including the heart tissue, skeletal muscle, and pulmonary system (Abdullah et al., 2018; Aishwarya et al., 2022b; Remex et al., 2023). To assess whether Sigmar1 plays a role in vascular remodeling, we have used different histological staining techniques using paraffin-embedded aortic root tissue section from Sigmar1^−/−^ mice and compared with that of Wt littermate controls. Hematoxylin and eosin (H&E), Masson’s trichrome, Russell-movat pentachrome, and toluidine blue staining of aortic root showed aberrant organization of smooth muscle cells in the medial layer compared to Wt mice (Figures 2A–D). The nuclei disarray of vascular smooth muscle layer and abrupt organization of elastic fibers was observed in the absence of Sigmar1 (Figure 2A). Masson’s trichrome (Figure 2B), Russell-movat pentachrome (Figure 2C), and Toluidine blue (Figure 2D) staining showed higher collagen deposition in the vascular layer and perivascular area (blue and yellowish red respectively) causing higher fibrotic remodeling in Sigmar1^−/−^ mice compared to Wt (Figures 2B–D). These staining images indicate that Sigmar1 has a potential role in vascular remodeling and vascular and perivascular fibrosis.

### 3.3 Enhanced aortic stiffness and impaired vascular function in absence of Sigmar1

**FIGURE 3:**
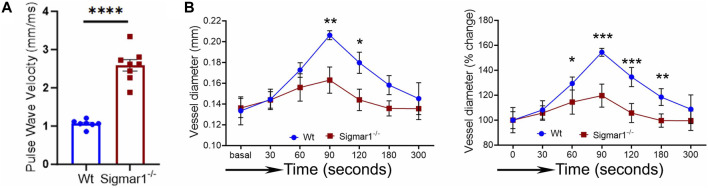
Sigmar1 deficiency causes increased pulse wave velocity (PWV) and decreased flow-mediated dilation (FMD) in mice. **(A)** Vascular stiffness was measured as PWV in the aorta of male Wt and Sigmar1^−/−^ mice at 3 months of age using echocardiogram. The transit time was identified from the M mode of pulse wave Doppler ultrasound velocity measurements, and the distance was measured from the B mode images from echocardiography data. The time-transit quantification for PWV identified significantly higher vascular stiffness in mice aorta in Sigmar1 null mice compared to Wt. N = 7 (Wt) and 8 (Sigmar1^−/−^). *p* values were determined by unpaired *t*-test. **(B)** Vascular function was measured by non-invasive FMD of left femoral artery in Wt and Sigmar1^−/−^ mice at 4 months of age using both male and female mice. The left femoral artery underwent transient ischemia for a minute, and then the trigger was removed. Arterial diameter was the highest at 90s post-ischemic timepoint, and FMD response gradually went back down close to the baseline. However, the Sigmar1^−/−^ mice showed significantly decreased FMD response indicating notable vascular dysfunction in response to reactive hyperemia. N = 5 mice per group (Wt; 3 male and 2 female mice) and 6 (Sigmar1^−/−^; 3 male and 3 female mice). The recorded loops were analyzed by Vevo LAB analysis software. Data were compared between groups using two-way ANOVA followed by Sidak’s multiple comparisons. A value of *p* < 0.05 was considered statistically significant. *p < 0.05; **p < 0.01; ***p < 0.001; ****p < 0.0001.

We next measured the *in vivo* pulse wave velocity (PWV) to determine vascular stiffness in the aorta of Wt and Sigmar1^−/−^ mice. We used 4 months-old male and female mice to measure PWV using doppler ultrasound probe (VEVO 3100). This is an indirect measurement of arterial stiffness in mice. The time-transit method of PWV analysis (DuPont et al., 2021) showed a significant increase of aortic stiffness in Sigmar1 deficient mice compared to Wt. This indicated more rigid blood vessels in Sigmar1^−/−^ mice (Figure 3A). Since Sigmar1 is expressed in the vascular wall and has a role in vascular remodeling, we then sought to examine the physiological function of Sigmar1 in the vascular system. To do so, we assessed vascular flow-mediated dilation (FMD) of the left femoral artery of Sigmar1^−/−^ mice and compared that to Wt mice. This is a gold standard non-invasive method in the vascular field to determine vascular function in response to reactive hyperemia. FMD measurements showed that transient hindlimb ischemia for 1 min with an occlusion cuff in Sigmar1^−/−^ mice did not induce femoral artery vasodilation upon reperfusion. The Wt mice showed FMD responses, especially at 90 s time point after removing the ischemic trigger, when the arterial diameter was the highest. Then, it gradually went back close to the baseline at 300 s time point. On the other hand, FMD responses were significantly blunted in Sigmar1 deficient mice (Figure 3B). Sigmar1^−/−^ mice failed to respond to reactive hyperemia after removing the ischemic trigger, indicating profound vascular dysfunction in response to brief tissue ischemia.

### 3.4 NO bioavailability and eNOS phosphorylation in Sigmar1^−/−^ mice aorta

**FIGURE 4:**
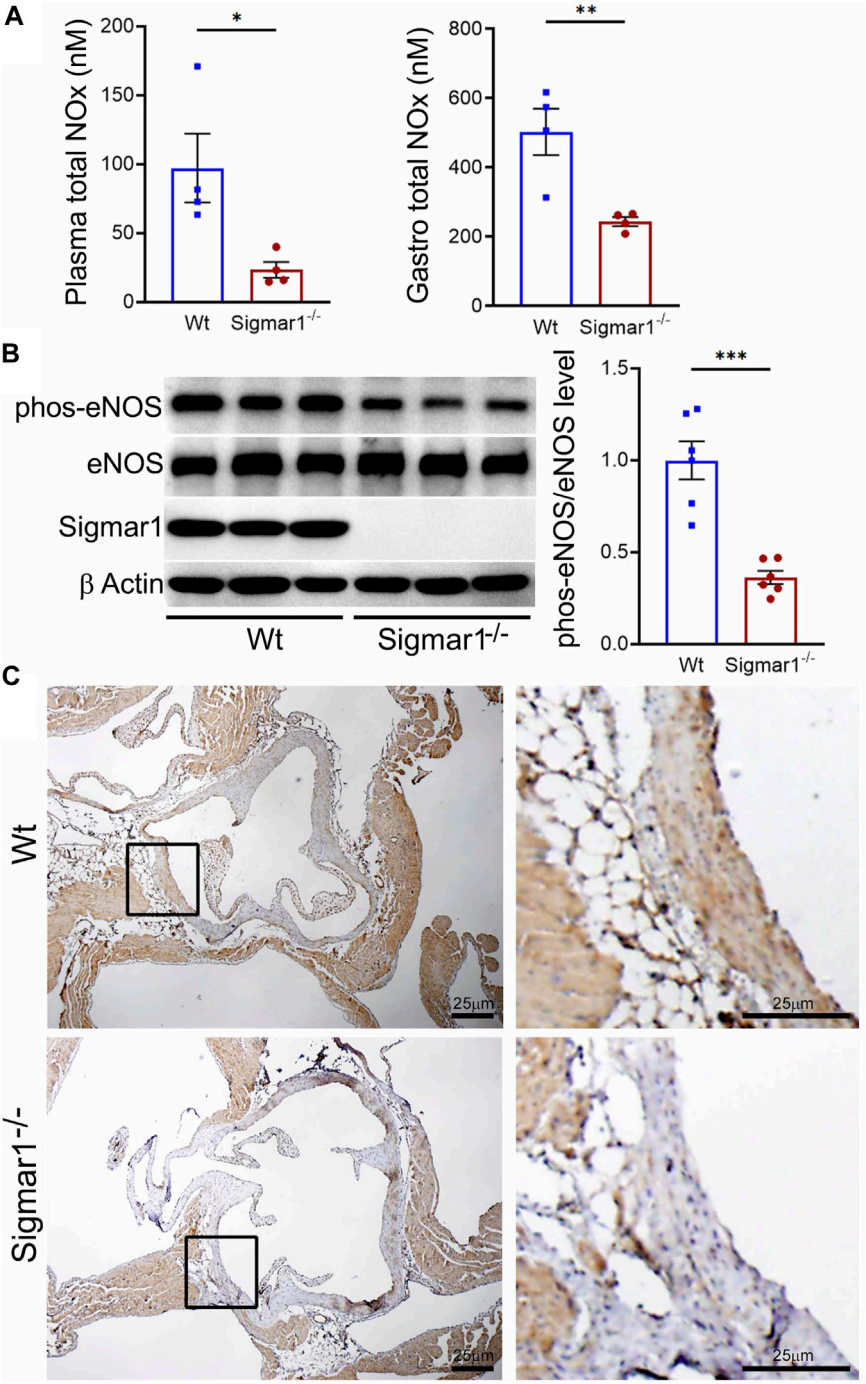
Sigmar1 deletion leads to decreased total nitric oxide metabolite (NOx) levels in the plasma and decreased phos-eNOS^ser1177^ protein levels in mice. Plasma, gastrocnemius muscle, aorta, and aortic roots were collected from 4 to 5 months-old Wt and Sigmar1^−/−^ mice. **(A)** Total NOx level was measured using a very sensitive ozone-based chemiluminescent assay in blood plasma and highly vascularized gastrocnemius muscle. Sigmar1 null mice showed a significant decrease in NOx level both in plasma and in the skeletal muscle compared to the Wt mice. N = 4 mice per group (2 male and 2 female mice). **(B)** Sigmar1 deficient mice also showed significant decrease in phos-eNOS^ser1177^ protein level in the aortic tissue lysate compared to that of Wt control mice. phos-eNOS^ser1177^ expression was normalized by total eNOS protein level. Sigmar1 Western blot confirmed complete deletion of the protein from Sigmar1^−/−^ mice aorta, and β-Actin was used as a housekeeping control. N = 6 mice per group; 3 male and 3 female mice. **(C)** Immunohistochemical staining of phos-eNOS^ser1177^ in aortic root was remarkably reduced in Sigmar1^−/−^ mice aortic roots. N = 3 mice per group; 2 male and 1 female mice. Scale bar 25 μm. *p* values were determined by non-parametric Mann-Whitney U and Kruskal-Wallis test. A value of *p* < 0.05 was considered statistically significant. *p < 0.05; **p < 0.01; ***p < 0.001.

Nitric oxide (NO) has been a key determinant of vascular homeostasis that has a wide variety of functions in the vasculature regulating the physiological properties of the blood vessels. It maintains vascular tone, permeability, and has anti-inflammatory and anti-thrombotic properties (Palmer et al., 1987). Most of the vascular NO is produced by the endothelial cell using nitric oxide synthase (eNOS) enzyme and exerts its effects on the vascular smooth muscle cells (vSMCs), maintaining vessel tone (Jin and Loscalzo, 2010). Decreased bioavailability of NO has been associated with many cardiovascular diseases, including hypertension, atherosclerosis, and diabetes mellitus (Forstermann et al., 2006) and is a determinant of vascular dysfunction. Therefore, we next measured the total bioavailability of NO in Wt and Sigmar1^−/−^ mice in the plasma and tissue using an ozone-based hypersensitive chemiluminescence assay. The plasma NO metabolites were significantly decreased in the absence of Sigmar1 compared to Wt mice at 4 months of age (Figure 4A). The total NOx level in the vascularized gastrocnemius muscle also showed a significant decrease in the Sigmar1^−/−^ mice (Figure 4A). Additionally, we observed a significant reduction of phos-eNOS^ser1177^ protein expression confirmed by Western blot of aorta tissue (Figure 4B) and immunohistochemical staining of aortic roots in Sigmar1^−/−^ mice compared to Wt (Figure 4C). This suggested depletion in NO synthesis and bioavailability in the vascular system in the absence of Sigmar1 indicating vascular dysfunction in mice.

### 3.5 TEM analysis of blood vessel wall of Sigmar1^−/−^ mice

**FIGURE 5:**
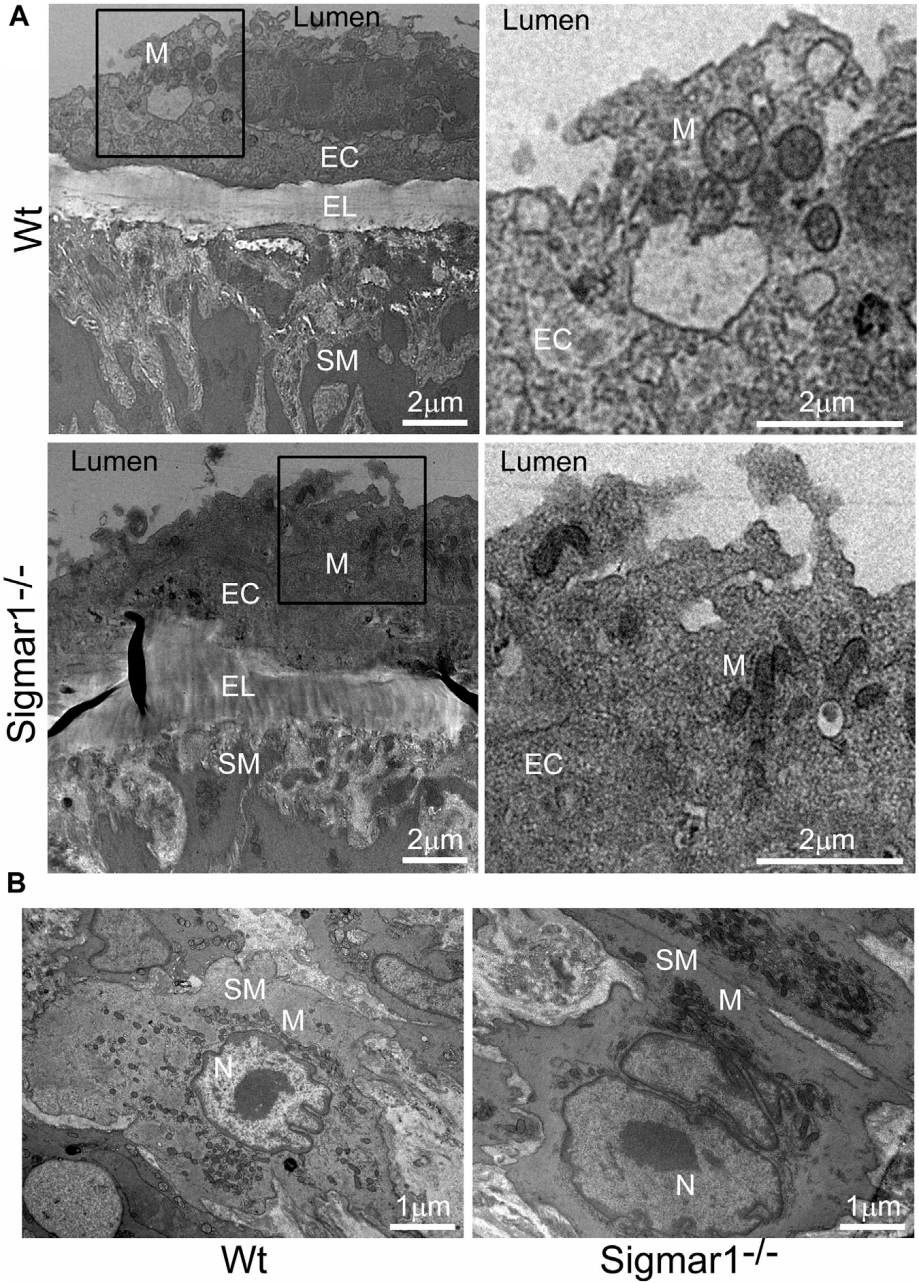
The ultrastructural analysis of mouse aortic tissue shows accumulation of elongated mitochondria in the vascular endothelial and smooth muscle cells in Sigmar1 null mice. Aorta tissues were collected from 4 months-old Wt and Sigmar1^−/−^ mice and analyzed for ultrastructural alterations using transmission electron microscopy (TEM). **(A)** TEM images of vascular endothelial cells in the aorta showed longer mitochondrial shapes and sizes in the absence of Sigmar1, whereas Wt endothelial cells showed normal, regular mitochondrial structure. Scale bar: 2 μm. **(B)** Representative TEM image of vascular smooth muscle cells from Sigmar1^−/−^ mice aorta showed accumulation of elongated mitochondria around the nucleus. Wt mice showed circular mitochondria with dispersed distribution in the aortic smooth muscle cell. N = 3 mice per group; 2 male and 1 female mice. Scale bar: 1 μm. EC: Endothelial cell, SM: Smooth muscle cell, EL: Elastic lemina, M: Mitochondria, N: Nucleus.

The aorta tissue isolated from Wt and Sigmar1^−/−^ mice was analyzed for ultrastructural morphology of vascular wall cells using transmission electron microscopy (TEM). The vascular wall comprises three distinct layers: tunica intima containing a monolayer of endothelial cells, tunica media containing multiple layers of vascular smooth muscle cells, and outer adventitia containing nerve endings, fibroblasts, adipose tissue, etc. TEM images of the mouse aorta showed a detailed visual representation of vascular endothelial and smooth muscle cells (Figure 5) in the artery. In both cell types, it is notable that the mitochondrial shape is altered in the absence of Sigmar1. The mitochondria become markedly more elongated and clustered in Sigmar1^−/−^ mice, which is consistent with some previous findings in cardiac and lung ultrastructure (Abdullah et al., 2018; Remex et al., 2023). In contrast, the Wt mouse aorta showed the rounder-shaped and dispersed distribution of mitochondria in the endothelial and smooth muscle cells. These results indicate that Sigmar1 has a role in regulating mitochondrial shape and structure in vascular cells.

### 3.6 Analysis of mitochondrial respirometry in *ex-vivo* aortic rings from Sigmar1^−/−^ mice using Oroboros

**FIGURE 6:**
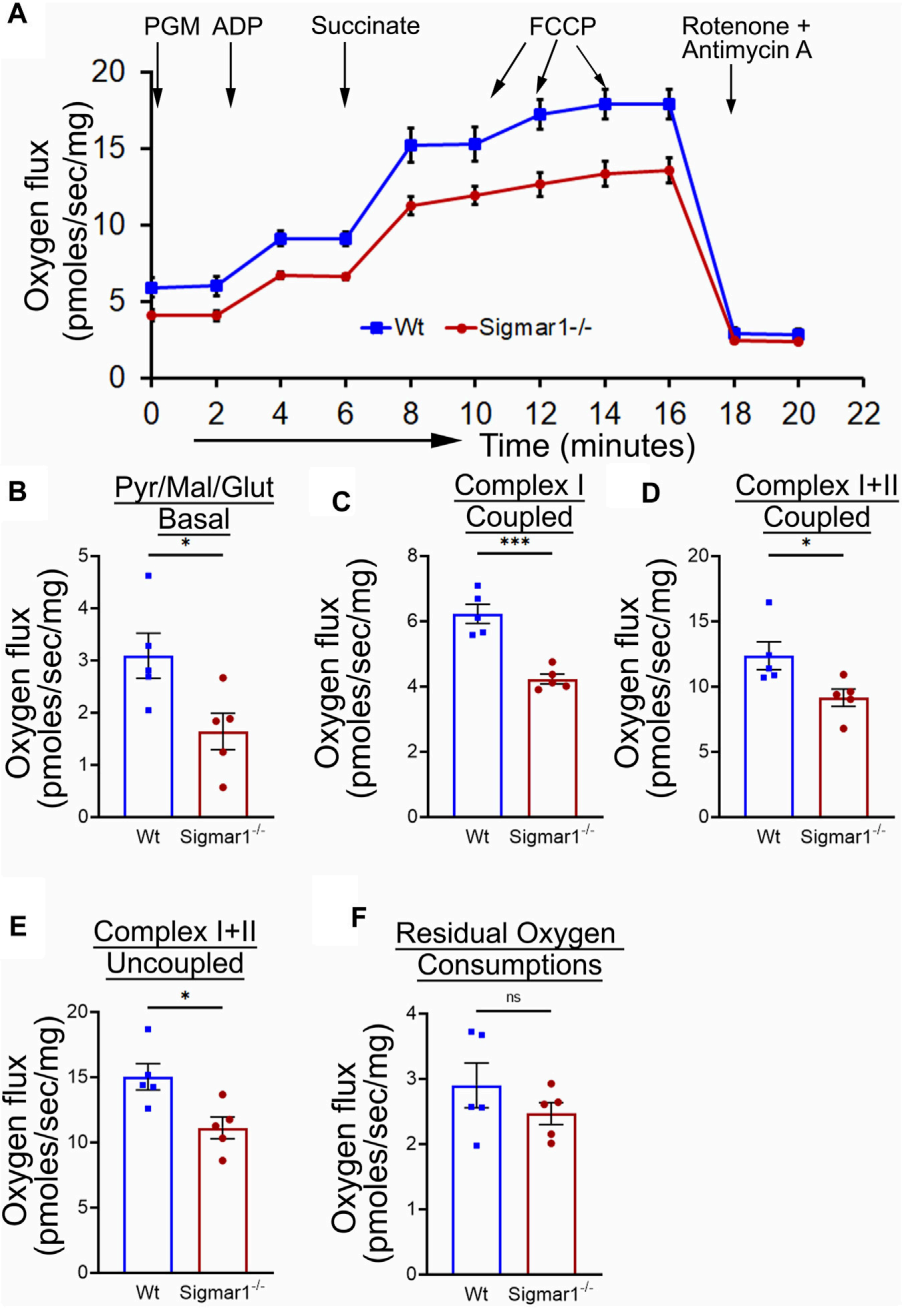
Sigmar1 deficiency results in reduced mitochondrial respiration and function in mouse aorta measured by high-resolution respirometry. Aorta tissues, including the aortic arch, thoracic, and abdominal aorta (until the bifurcation of iliac arteries) were isolated from 3 months-old male and female Wt and Sigmar1^−/−^ mice. The aortas were immediately cut into small rings of 2–3 mm and placed in the oxygraph chamber to measure mitochondrial oxygen flux in *ex-vivo* aortic rings. Different inhibitors, substrates, and uncouplers were used to measure oxygen flux of mitochondrial respiratory chain complexes in high-resolution respirometry. **(A)** The mitochondrial oxygen flux of Sigmar1^−/−^ mice aortic rings was substantially decreased compared to Wt mice aortas. **(B–F)** oxygen flux at the basal, Complex I coupled, complex I + II coupled, and Complex I + II uncoupled states were significantly reduced in the aortic rings from Sigmar1^−/−^ mice compared to Wt mice. The residual oxygen consumption rate was comparable between the two groups. All oxygen flux values were normalized by the wet weights of the aorta tissue. N = 5 mice per group; 3 male and 2 female mice. *p* values were determined by the Mann-Whitney U test. A value of *p* < 0.05 was considered statistically significant. *p < 0.05; **p < 0.01; ***p < 0.001; ns, not significant.

Mitochondrial structural alteration is an indication of functional impairment as well, which is a hallmark of different cardiovascular diseases (Abdullah et al., 2018; Alam et al., 2018; Tyrrell et al., 2020; Abdullah et al., 2022b). Since Sigmar1^−/−^mice showed elongated and clustered mitochondria in aortic endothelial cells and smooth muscle cells, we measured mitochondrial function using high-resolution mitochondrial respirometry in aortic rings from Wt and Sigmar1^−/−^ mice (Figure 6). To determine mitochondrial fuel substrates-supported respiration in aortic rings, we injected NADH-generating substrates (donate electrons to complex I, CI), i.e., pyruvate, malate, and glutamate, followed by ADP addition to attain CI-supported oxidative phosphorylation (OXPHOS) state. Next, we injected succinate (activates complex II, CII) to measure respiration at the CI + CII-coupled OXPHOS state. FCCP was injected to uncouple mitochondrial respiration from OXPHOS to measure CI + CII-linked uncoupled respiration. Mitochondrial respiratory complex I inhibitor (Rotenone) and complex III inhibitor (Antimycin A) were injected at the end to measure non-mitochondrial residual oxygen flux (Figure 6A). These respirometry study protocol revealed significantly attenuated mitochondrial complex I supported basal respiration with impaired complex I + complex II-linked coupled and uncoupled respiration with non-significant changes in residual nonmitochondrial respiration in Sigmar1^−/−^ mice aorta compared to Wt mice aorta (Figures 6B–F). This finding indicate that Sigmar1 is essential to maintain mitochondrial respiration in the vascular system.

## 4 Discussion

**FIGURE 7:**
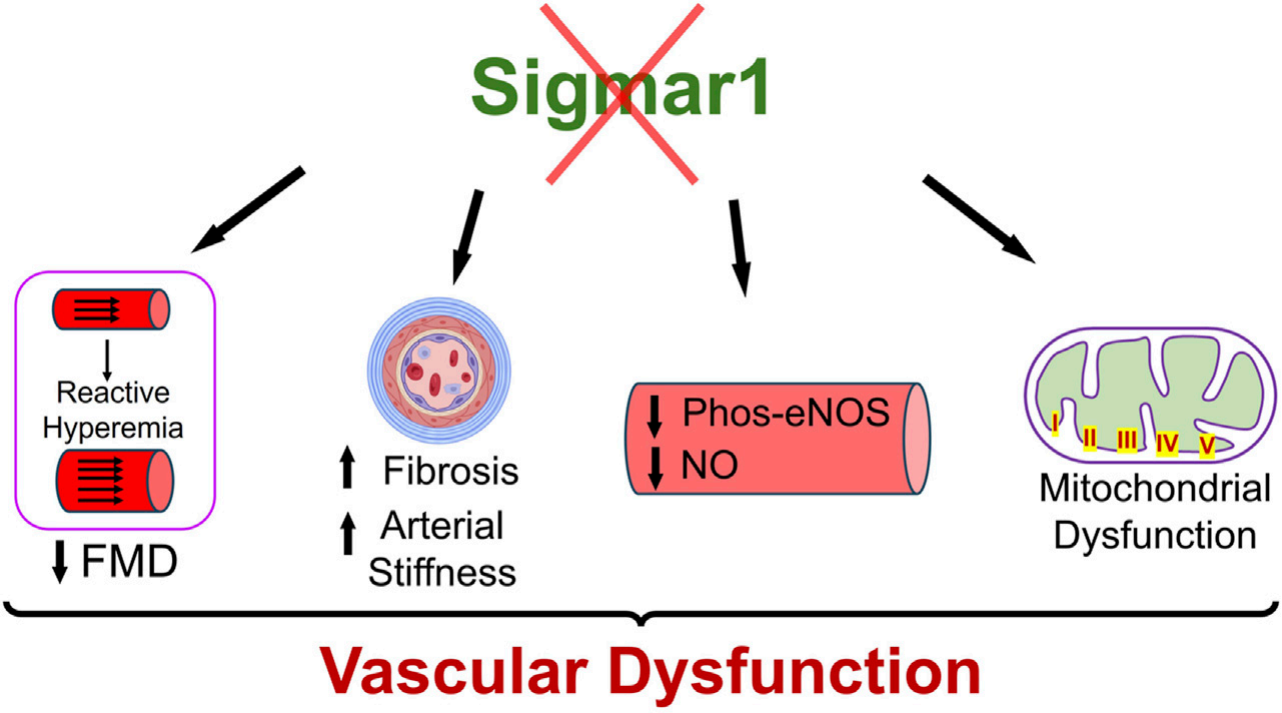
Schematic diagram showing how Sigmar1 deficiency leads to vascular dysfunction. Absence of Sigmar1 contributes to decreased flow mediated dilation, decreased eNOS phosphorylation and nitric oxide bioavailability, increased vascular fibrosis and aortic stiffness in mice. This also leads to altered mitochondrial shape and structure causing mitochondrial dysfunction in global Sigmar1 mice.

Sigma1 receptor (Sigmar1) is a multifunctional chaperone protein with important molecular and cellular functions, including ion channel regulation, lipid transportation, mitochondrial functions, and autophagy (Aishwarya et al., 2021). It plays a vital role in maintaining cardiovascular homeostasis by protecting hearts during heart failure and cardiac ischemia-reperfusion injury and regulating cardiac contractility and mitochondrial functions (Abdullah et al., 2018). Given the significant neuroprotective and cardioprotective roles of Sigmar1 that have been studied, the physiological roles of this protein in the vascular system remain elusive. This study confirmed its expression in the human and mouse arteries using histological and biochemical techniques. Functional assays indicated that global loss of Sigmar1 in mice leads to stiffer blood vessels, impaired response to reactive hyperemia upon the ischemic trigger, and decreased flow-mediated dilation (FMD) in the left femoral artery. This was consistent with decreased nitric oxide bioavailability and eNOS phosphorylation in global Sigmar1 deficient mice (Sigmar1^−/−^) compared to wildtype controls (Wt). The Sigmar1^−/−^ mice also showed vascular reorganizations, altered mitochondrial structures, and decreased mitochondrial respiration in aorta. These findings indicate that Sigmar1 is a potential player in maintaining vascular homeostasis and mitochondrial function in the vascular tissue (Figure 7).

Sigmar1 was first identified in the thoracic aorta in a hypertrophy-induced vascular injury model of ovariectomized rats where the protein level of Sigmar1 was significantly decreased in the pressure overload-induced injured blood vessels (Bhuiyan et al., 2011b). Later, a few studies detected Sigmar1’s protein expression levels using Western blot analysis in the lymphatic endothelial cells (Motawe et al., 2021b) and human brain endothelial cells (Liu et al., 2022) to determine its protective role in endothelial barrier function. However, these ligand-dependent studies failed to show the protein expression level and distribution of Sigmar1 in vascular tissue using histological or immunostaining. We hereby identified Sigmar1 protein expression in both human and mouse vascular tissue using immunohistochemical and immunofluorescence staining (Figure 1). Sigmar1 protein is substantially expressed in all three vascular layers (innermost intima, middle smooth muscle cell layer media, and the outermost adventitia) in human left anterior descending coronary artery (LAD) and mouse aortic root. Sigmar1 protein expression is also detectable in the aorta in mice using Western blot. This study is the first to show this protein’s expression level and distribution in the vascular tissue in humans and mice.

Loss of Sigmar1 led to adverse cardiac remodeling, increased perivascular and interstitial collagen deposition, and increased fibrotic markers in the heart in our previous study (Abdullah et al., 2018). This evidence indicates that Sigmar1 has a potential role in tissue remodeling, resulting in significant fibrosis and collagen deposition. In this study, we observed remarkable vascular fibrosis in the aortic roots of Sigmar1^−/−^ mice compared to Wt (Figure 2). We also showed reorganization of nuclear disarray in the medial layer, which is mostly vascular SMCs along with abrupt arrangement of elastic lamina in the aortic roots of Sigmar1^−/−^ mice compared to Wt (Figure 2). This was consistent with our previous findings indicating Sigmar1^−/−^ mice develop fibrotic remodeling in the heart, lung, and skeletal muscle (Abdullah et al., 2018; Aishwarya et al., 2022b; Remex et al., 2023). Vascular fibrosis and extracellular matrix remodeling have been associated with many vascular pathologies, such as hypertension, atherosclerosis, and peripheral artery disease (PAD) (Lan et al., 2013; Harvey et al., 2016; Ding et al., 2020). Hypertension is closely associated with increased ECM content, especially the fibrillar collagen type, leading to vessel stiffness over time (Bashey et al., 1989). As Sigmar1^−/−^ mice reported to have altered hemodynamics (Abdullah et al., 2018), which might be partially contributing to the ECM remodeling and higher collagen content in the vascular tissue in absence of Sigmar1.

Since ECM remodeling, collagen deposition, and vascular tissue reorganization are leading causes of vessel stiffness, it was worthy of measuring vascular stiffness in Sigmar1^−/−^ mice. Using non-invasive pulse wave velocity (PWV), we determined the aortic stiffness indirectly following the previously used time-transit method (Williams et al., 2007; DuPont et al., 2021). Sigmar1 deficient mice showed significantly higher aortic stiffness compared to Wt (Figure 3). Consistently, vascular functional analysis showed a decline in flow-mediated dilation (FMD) in left femoral artery in response to ischemic trigger indicating vascular dysfunction in absence of Sigmar1 (Figure 3). The Sigmar1^−/−^ mice failed to increase vessel diameter in response to reactive hyperemia and this can be associated with stiffer blood vessels in these mice. FMD is also a non-invasive indirect assessment of endothelial function and can be used to examine endothelial dysfunction, which is an early event in the initiation of many vascular diseases like atherosclerosis (Matter et al., 2011). A decrease in FMD provides an index of vascular endothelium-derived nitric oxide (NO) function. It can indicate either insufficient production of endothelial NO or improper response of vascular SMCs to NO, leading to dysfunction in vessel dilation (Green et al., 2011). In line with this evidence, we also observed a significant decline in NO bioavailability in Sigmar1^−/−^ mice compared to Wt controls (Figure 4). NO is an essential vasodilator molecule that regulates vascular tone, maintains vessel resistance, and overall vascular homeostasis. Decline in NO level can lead to stiffer vessel resistance and vascular dysfunction. Vascular NO is synthesized by the endothelial cells with the help of endothelial nitric oxide synthase enzyme (eNOS). Activation of Sigmar1 using its pharmacological agonist has previously been shown to have a protective effect on hypertrophy-mediated vascular injury in rats by upregulating AKT-eNOS signaling (Bhuiyan et al., 2011a; Bhuiyan et al., 2011b). When this signaling axis is activated in the vasculature, PI3K signaling pathway phosphorylates the AKT, which then phosphorylates eNOS at serine 1177, and synthesizes NO. In line with previous data, we observed a decrease in phos-eNOS^ser1177^ level in the aorta lysate and aortic root tissue (Figure 4), indicating dysfunction in NO synthesis pathway.

Sigmar1 has long been known to regulate mitochondrial structure and functions in brain cells, cardiomyocytes, and skeletal muscles (Bernard-Marissal et al., 2015; Abdullah et al., 2018; Aishwarya et al., 2021; Aishwarya et al., 2022b). Sigmar1 is a mitochondrial membrane protein regulating mitochondrial respiration, dynamics, bioenergetics, and architecture (Alam et al., 2018; Abdullah et al., 2022a; Aishwarya et al., 2022a). Deletion of Sigmar1 globally in mice led to the accumulation of elongated shaped mitochondria, defective mitochondrial respiratory function, and altered mitochondrial dynamics in the heart, leading to impairment in ATP generation and heart contractile dysfunction (Abdullah et al., 2018). Our ultrastructural analysis of the aorta section showed accumulation of irregularly longer shaped mitochondria in aortic endothelial and smooth muscle cells in Sigmar1^−/−^ mice (Figure 5). Analysis of mitochondrial respirometry parameters in *ex-vivo* aortic rings showed significant reduction in mitochondrial oxidative phosphorylation-linked respiration in absence of Sigmar1 (Figure 6). Mitochondrial dysfunctions are strongly associated with cardiovascular diseases, for example, atherosclerosis, ischemic heart disease, hypertension, and cardiomyopathy (Forte et al., 2021; Ciccarelli et al., 2023). Mitochondrial dynamics-related abnormalities can lead to the development of cardiovascular diseases (Hubens et al., 2022). Impaired mitochondrial function causes a reduction in ATP production, generation of reactive oxygen species (ROS) and related signaling, apoptosis and cell survival, dysfunctional electron transport chain activities, and trigger inflammatory responses (Iglewski et al., 2010; Lee et al., 2016; Luongo et al., 2017; Zhou and Tian, 2018). Mitochondrial electron transport chain complexes being the major sources of ROS generation, especially, complex I and II, dysfunction of the mitochondrial respiratory system can lead to an excess burden in mitochondrial and overall cellular oxidative stress (Madamanchi and Runge, 2007). Excessive ROS production can cause damage in mitochondrial DNA and oxidation of important proteins, lipids, and enzymes leading to mitochondrial and cellular dysfunction (Peng et al., 2019). Dysfunctional mitochondria, higher ROS, and reduced NO bioavailability can also signal for release of inflammatory cytokines and chemokines causing activation of inflammatory pathways (Qu et al., 2022; Ciccarelli et al., 2023). These are underlying leading factors for many inflammatory and metabolic cardiovascular diseases like atherosclerosis. Thus, mitochondrial homeostasis is crucial for cardiovascular health, and Sigmar1, being an important player in regulating mitochondrial respiration and function, can be an attractive therapeutic target for mitochondrial dysfunction-related cardiovascular pathologies.

A limitation of the present study is the use of Sigmar1 global knockout mice in which the loss of Sigmar1 in other organs may also contribute to observed vascular dysfunction. In fact, hemodynamics measurements in the Sigmar1^−/−^ mice in our previous study showed higher systolic blood pressure, mean arterial pressure, and left ventricular pressure compared to Wt mice (Abdullah et al., 2018). However, significant alterations in arterial stiffness, flow-mediated dilation, and vascular structural changes in the aorta of Sigmar1^−/−^ mice demonstrate a potential pathophysiological role of Sigmar1 which should be delineated using both endothelial cell and smooth muscle cell specific Sigmar1 knockout mice.

## 5 Conclusion

This study has identified Sigmar1 protein expression level and distribution in the human and mouse arteries. Using Sigmar1 global knockout and wildtype control mice, we found the development of vascular dysfunction in the absence of Sigmar1 protein (Figure 7). Loss of Sigmar1 led to vascular reorganizations, ECM remodeling, increased arterial stiffness, and decreased flow-mediated dilation. This was consistent with a significant decline in total nitric oxide bioavailability in plasma and tissue and decreased phos-eNOS^ser1177^ expression in aortic lysate. Finally, the ultrastructural analysis of aortic tissue showed an accumulation of elongated mitochondria in the vascular cells and decreased mitochondrial respirometry parameters in *ex-vivo* aortic rings from Sigmar1 deficient mice compared to Wt controls.

## Data Availability

The raw data supporting the conclusion of this article will be made available by the authors, without undue reservation.
