# Supplementary figures and images for: Deletion of Sigmar1 leads to increased arterial stiffness and altered mitochondrial respiration resulting in vascular dysfunction
Source: Front Physiol. 2024 Apr 29;15:1386296. doi: 10.3389/fphys.2024.1386296 (PMC11089145; doi:10.3389/fphys.2024.1386296)

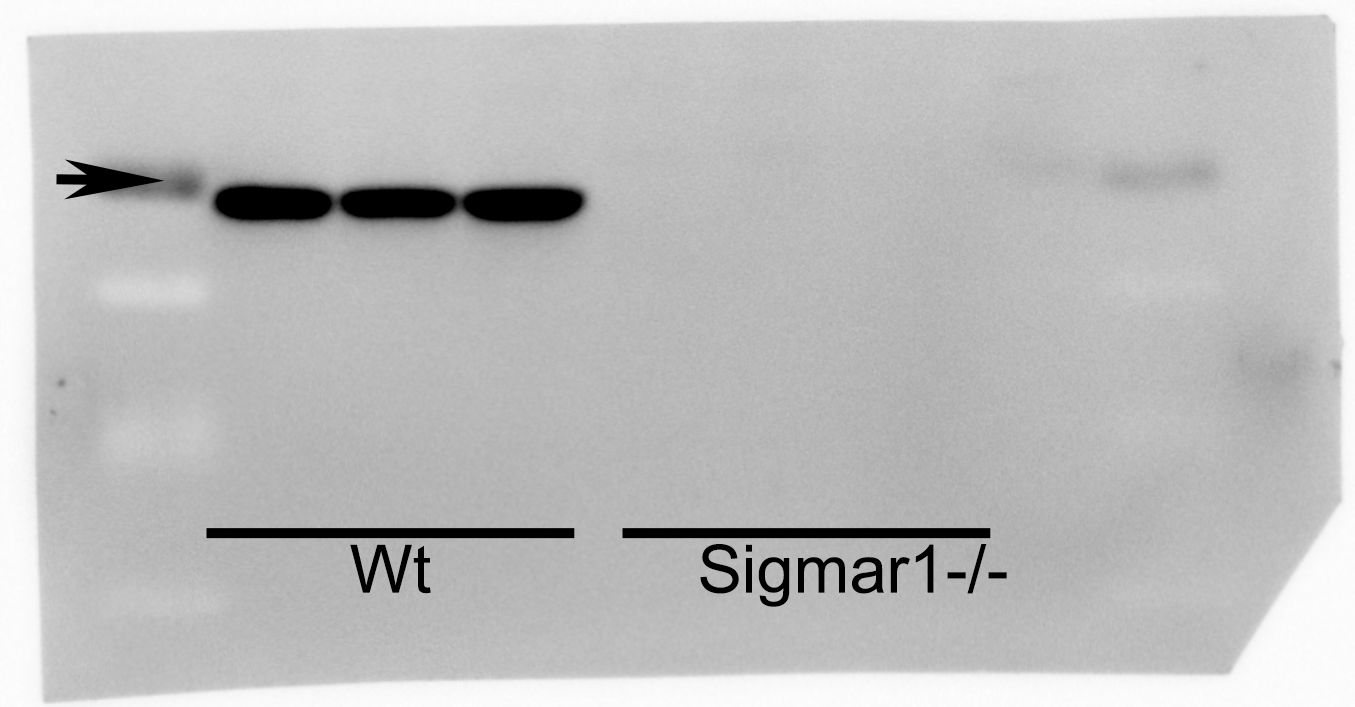

Supplement: Supplementary file 1 [file Image3.TIF]

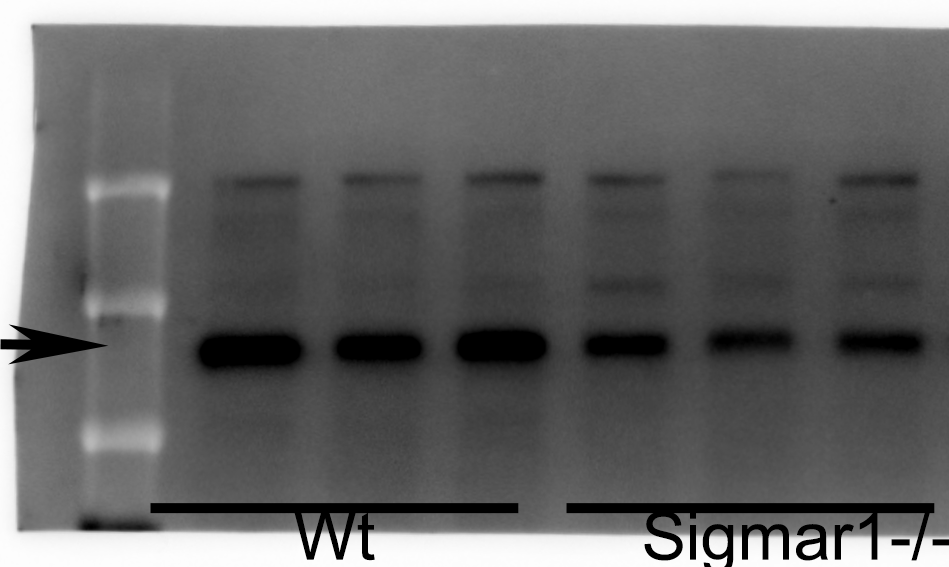

Supplement: Supplementary file 2 [file Image4.TIF]

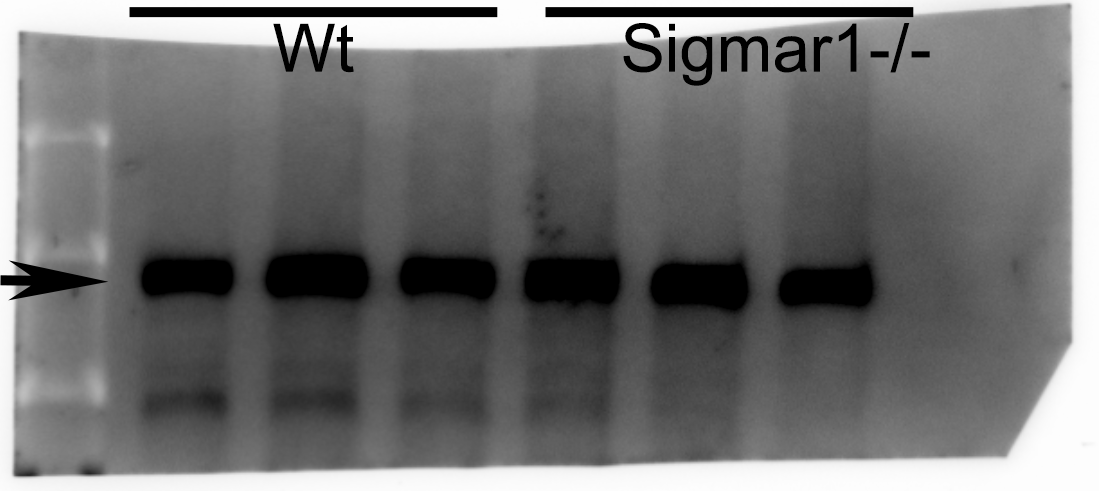

Supplement: Supplementary file 3 [file Image2.TIF]

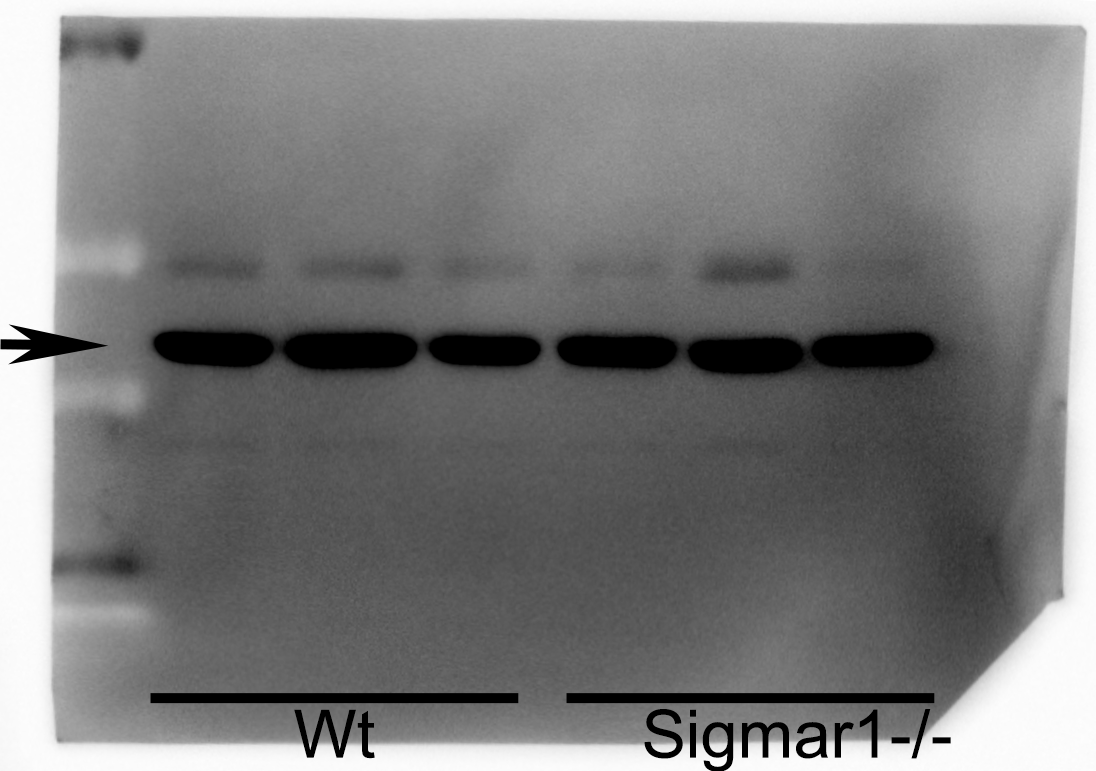

Supplement: Supplementary file 4 [file Image1.TIF]
